# Meeting report on the Alzheimer’s Drug Discovery Foundation 14th International Conference on Alzheimer’s Drug Discovery

**DOI:** 10.1186/alzrt252

**Published:** 2014-04-28

**Authors:** Lauren G Friedman, Katherine Price, Rachel F Lane, Aaron J Carman, Penny A Dacks, Diana W Shineman, Howard M Fillit

**Affiliations:** 1 Columbia University, Taub Institute, 630 West 168th Street, New York, NY 10032, USA; 2 Icahn School of Medicine at Mount Sinai, 1470 Madison Avenue, New York, NY 10029, USA; 3 Alzheimer’s Drug Discovery Foundation, 57 West 57th Street, Suite 904, New York, NY 10019, USA

## Abstract

The Alzheimer’s Drug Discovery Foundation’s 14th International Conference on Alzheimer’s Drug Discovery was held on 9 and 10 September in Jersey City, NJ, USA. This annual meeting highlights novel therapeutic approaches supported by the Alzheimer’s Drug Discovery Foundation in development for Alzheimer’s disease and related dementias.

## Introduction

Alzheimer’s disease (AD) is a complex, multifactorial disease with an urgent need for effective, disease-modifying treatments. Researchers continue to make progress on promising targets that contribute to mitochondrial dysfunction, altered lipid and cholesterol metabolism, neuroinflammation, and synaptic dysfunction. Presenters at the 2013 International Conference on Alzheimer’s Drug Discovery emphasized diverse preclinical and clinical therapeutic approaches.

In the first of two plenary sessions, Nir Barzilai (Albert Einstein College of Medicine, Bronx, NY, USA) explored longevity-associated genotypes in centenarians to identify therapeutic targets for cognitive decline. DNA sequencing of blood collected from centenarians with preserved cognitive function identified coding variants in insulin growth factor-1 and cholesteryl ester transfer protein, reinforcing the contribution of both metabolism and cholesterol homeostasis to cognitive function. A phase III trial of a cholesteryl ester transfer protein inhibitor (Merck & Co, NJ, USA) is underway for cardiovascular disease, but may show promise for the prevention of AD.

In the second plenary session, Jeff Cummings (Cleveland Clinic, Cleveland, OH, USA) highlighted the advantages and challenges of repurposing drugs approved for other diseases to the treatment of AD. While the availability of preclinical data, human safety data, and pharmacokinetic data may shorten timelines, there are significant costs and risks associated with testing the safety and efficacy of a repurposed drug for a new indication in phase II and phase III clinical trials. Significant commercial challenges relating to intellectual property also exist. Despite these challenges, a variety of approved drugs for oncology, diabetes, hypertension, cholesterol, and neurodegenerative movement disorders, among others, have been shown to have AD-relevant effects. Several proposed repurposed drugs for AD that are currently in phase II trials are highlighted in the repurposing section.

## Neuroprotection and neuroinflammation

Small peptide fragments of neuronal growth factors are a promising neuroprotective strategy. Joseph Harding (M3 Biotechnology, Seattle, WA, USA) described the development of dihexia, a novel blood–brain barrier (BBB)-permeable hepatocyte growth factor mimetic derived from angiotensin IV. Dihexia potentiates the activity of hepatocyte growth factor at the tyrosine kinase receptor, cMet, rescuing behavioral deficits in a scopolamine model and in a Parkinson’s disease lesion model. Antonio Catteneo (Scuola Normale Superiore, Pisa, Italy) demonstrated that intranasal administration of human nerve growth factor (hNGF P61S/R100E) rescued cognitive deficits, reduced amyloid plaque load and size, and lowered amyloid-beta (Aβ) levels without causing hyperalgesia in the APP/PS1 mouse model. Khalid Iqbal (New York State Institute for Basic Research, New York City, NY, USA) discussed peptide-6, a peptide fragment of the ciliary neurotrophic factor that decreased Aβ and phospho-tau in a mouse model of AD.

Paolo Pevarello (Axxam SpA, Milan, Italy) discussed a small-molecule approach to target neuroinflammation through modulation of microglial ligand-gated receptor P2X_7_, which is required for microglial activation by Aβ [1]. While P2X_7_ antagonists are used in the clinic for rheumatoid arthritis, Pevarello presented lead optimization data for novel compounds that are central nervous system penetrant. John Schetz (University of North Texas Health Science Center, Fort Worth, TX, USA) presented lead optimization data for modulators of sigma-1 receptor. Activation of sigma-1 receptor selectively inhibits inducible nitric oxide synthase activity at sites of inflammation. Seema Briyal (Midwestern University, Downers Grove, IL, USA) discussed a preclinical program targeting the endothelin-B receptor, expressed by both endothelial cells and astrocytes. IRL-1620, a novel endothelin-B receptor agonist, increased expression of vascular endothelial growth factor receptor and nerve growth factor to stimulate angiogenesis and neurogenesis in an ischemic rat model.

To compensate for transcriptional shifts that have been described in several neurodegenerative diseases, Tamara Maes (Oryzon Genomics, Barcelona, Spain) presented the rationale for ORY-2001, a new BBB-permeable, histone demethylase LSD1/monoamine oxidase-B inhibitor. ORY-2001 restored behavioral deficits and protein levels of ubiquitin carboxyl-terminal esterase L1 (UCHL1) and Notch1 in the SAMP8 mouse model of accelerated senescence. The Oryzon dual LSD1/monoamine oxidase-B inhibitor is ready for pre-investigational new drug-enabling studies for AD.

Lawrence Wennogle (Intra-cellular Therapies, Inc., New York, NY, USA) described the development of novel therapeutics that target overexpression of casein kinase-1, a circadian rhythm protein that may cause sleep disruption in AD patients, and has been linked to enhanced Aβ production and tau phosphorylation. Intra-cellular Therapies, Inc., has identified selective, BBB-permeable lead casein kinase-1 inhibitors that decrease hyperlocomotion in a casein kinase-1-overexpressing mouse model, and may have therapeutic potential for AD.

## Glucose metabolism and mitochondrial dysfunction

Impaired glucose metabolism is a key feature of AD progression. Targeting mitochondrial dynamics, Eugenia Trushina (Mayo Clinic, Rochester, MN, USA) presented data on CP2, a tricyclic pyrone that rescued cognitive and motor deficits in three AD mouse models. Trushina’s metabolomics approach revealed that CP2 restored mitochondrial dysfunction rather than directly affecting Aβ formation. CP2 analogs for lead optimization are currently being developed.

Jerry Colca (Metabolic Solutions Development Company, Kalamazoo, MI, USA) presented clinical data on MSDC-1060, a novel thiazolidinedione. MSDC-1060 was engineered to spare peroxisome proliferator-activated receptor-gamma activity to bypass side effects associated with thiazolidinediones. In a small phase IIa study of mild to moderate AD patients, the fludeoxyglucose positron emission tomography signal in patients treated with MSDC-1060 decreased less than placebo-treated when referenced to the cerebellum.

## Repurposing US Food and Drug Administration-approved drugs for Alzheimer’s disease

Ana Pereira (Rockefeller University, New York, NY, USA) presented the rationale for a pilot study of riluzole, a glutamate modulator approved to treat amyotrophic lateral sclerosis. The study will test riluzole in mild cognitive impairment (MCI) patients, with magnetic resonance spectroscopy, arterial spin labeling, and fludeoxyglucose positron emission tomography as the primary outcomes.

In addition to the glutamatergic system, the noradrenergic system may impact disease progression. MCI and AD patients exhibit a loss of noradrenergic neurons and decreased norepinephrine. Allan Levey (Emory University, Atlanta, GA, USA) presented the rationale for atomoxetine, a norepinephrine transporter inhibitor approved for attention-deficit/hyperactivity disorder to increase norepinephrine levels. Preclinical studies demonstrated that treatement with atomoxetine plus lDOPS, a synthetic precursor in norepinephrine biosynthesis, increased norepinephrine, nerve growth factor brain-derived neurotrophic factor levels, decreased amyloid plaque load, and reversed behavioral deficits in the 5xFAD model. A phase II study in subjects with MCI is underway, measuring plasma and cerebrospinal fluid (CSF) markers of inflammation and CSF Aβ and tau.

Type 2 diabetes mellitus may increase the risk of AD. Jose Luchsinger (Columbia University Medical Center, New York, NY, USA) presented data that metformin improved insulin resistance and performance on a spaced retrieval test in a small pilot study of MCI patients. Paul Edison (Imperial College London, UK) presented his rationale for a phase IIa clinical trial underway with liraglutide, a glucagon-like peptide-1 analog that modulates insulin signaling, and has been shown to increase synaptic plasticity, decrease soluble Aβ, and improve behavior in AD mouse models.

Mid-life hypertension may also raise the risk of AD and vascular and mixed dementias, suggesting that anti-hypertensive treatments may protect against dementia. Sandra Black (University of Toronto, ON, Canada) presented the rationale for an open label, proof-of-concept trial that tests the effects of sartans on brain atrophy in hypertensive mild to moderate AD patients. The study compares the effects of telmisartan, an angiotensin receptor blocker with the longest half-life and greatest brain penetration of its class, and perindropil, an angiotensin-converting enzyme inhibitor that reduces dementia from stroke, but has not been shown to improve nonvascular dementia.

## Translatable biomarkers to accelerate clinical development

Blood-based biomarkers may help to detect AD prior to clinical symptoms, predict progression, differentiate AD from other neurodegenerative diseases, and quantify response to treatment. Michelle Mielke (Mayo Clinic) discussed sphingolipid metabolites as biomarkers to differentially diagnose AD and Lewy-body dementia. In a pilot study, high sphingomyelin levels predicted an increased risk of developing AD while plasma levels of glucosylceramide predicted Lewy-body dementia disease severity. Ongoing studies will further characterize sphingolipid levels in relation to disease pathology.

Douglas Galasko (University of California, San Diego, CA, USA) presented data that neural pentraxins, which regulate AMPA-receptor trafficking, are detectable in CSF and correlate with cognitive impairment, possibly reflecting changes in glutamatergic receptor clustering in response to AD-related alterations in synaptic activity. Marcie Glicksman (Harvard Neurodiscovery Center, Boston, MA, USA) discussed methods to stratify patients based on their mitophenotype as a peripheral biomarker for clinical testing of compounds that target this pathological mechanism.

## Apolipoprotein E

Apolipoprotein E (APOE)4 is the strongest genetic risk factor for late-onset AD, while APOE2 may be protective. Steve Paul (Weill Medical College of Cornell University, New York, NY, USA) presented data that adeno-associated virus-mediated gene delivery of APOE2 resulted in sustained ApoE2 expression in the hippocampus and decreased Aβ in the PDAPP mouse model. Thomas Wisniewski (New York University, New York, NY, USA) presented evidence that peptide inhibitors of the ApoE–Aβ interaction decreased fibrillar amyloid deposits, memory deficits, and cerebral amyloid angiopathy in the Tg2576 and APP/PS1 mouse models. Steve Estus (University of Kentucky Research Foundation, Lexington, KY, USA) presented a biomarker study to assess the effects of valproic acid on CSF ApoE–Aβ complexes and clusterin. Valproic acid, an approved histone deacetylase inhibitor, has been demonstrated to decrease Aβ, improve behavioral deficits in transgenic mice, and possibly modulate ApoE expression.

## Tau and protein clearance

Epigenetic modification of tau may provide an effective therapeutic strategy for tauopathies [2]. Tae-Wan Kim (Columbia University Medical Center, New York, NY, USA) presented a screen of small molecules that reduced tau protein levels in embryonic stem cell-derived neurons from hTau mice. A lysine-specific demethylase inhibitor was identified that reduced tau levels and restored Aβ-induced tau mislocalization, and ongoing studies will test the most promising leads in Tg2576 and P301S mouse models.

Protein clearance pathways provide an opportunity to stimulate clearance of tau and Aβ deposits. Li Huang (Duke University, Durham, NC, USA) presented novel proteasomal activators that reverse the inhibitory effects of Aβ oligomers on proteasomal clearance. LAD39 and DC139, derivatives of betulinic acid, enhance proteasome activity and clearance of Aβ and tau. Haung Yu (Columbia University) discussed approaches to promote autophagy-driven tau clearance. Yu presented data on a BBB-permeable derivative of quinazolinone, a potent inhibitor of huntingtin protein aggregation that might induce autophagy and tau clearance.

## Closing remarks

AD is caused by numerous underlying factors, so a diverse portfolio of promising drug discovery and clinical development programs is underway. For information on the 15th International Conference on Alzheimer’s Drug Discovery on 8 and 9 September 2014, please visit the World Events Forum website [3]. For more information on other Alzheimer’s Drug Discovery Foundation scientific events, visit the Foundation’s website [4].

## Abbreviations

AD: Alzheimer’s disease; apoE: Apolipoprotein E; Aβ: Amyloid-beta; BBB: Blood–brain barrier; CSF: Cerebrospinal fluid; MCI: Mild cognitive impairment.

## Competing interests

The authors declare that they have no competing interests.
